# Maternal Methyl-Group Donor Intake and Global DNA (Hydroxy)Methylation before and during Pregnancy

**DOI:** 10.3390/nu8080474

**Published:** 2016-08-06

**Authors:** Sara Pauwels, Radu Corneliu Duca, Roland Devlieger, Kathleen Freson, Dany Straetmans, Erik Van Herck, Inge Huybrechts, Gurdun Koppen, Lode Godderis

**Affiliations:** 1 KU Leuven, Department of Public Health and Primary Care, Environment and Health, Kapucijnenvoer 35 blok D box 7001, 3000 Leuven, Belgium; radu.duca@kuleuven.be (R.C.D.); lode.godderis@med.kuleuven.be (L.G.); 2 Flemish Institute of Technological Research (VITO), Unit Environmental Risk and Health, Boeretang 200, 2400 Mol, Belgium; gudrun.koppen@vito.be; 3 KU Leuven, Department of Development and Regeneration, 3000 Leuven, Belgium; Roland.devlieger@med.kuleuven.be; 4 Department of Obstetrics and Gynecology, University Hospitals of Leuven, 3000 Leuven, Belgium; 5 KU Leuven, Center for Molecular and Vascular Biology, UZ Herestraat 49-box 911, 3000 Leuven, Belgium; Kathleen.Freson@med.kuleuven.be; 6 AML Laboratory, Department of Endocrinology, 2000 Antwerp, Belgium; dany.straetmans@aml-lab.be; 7 KU Leuven, Unit Clinical and Experimental Endocrinology, UZ Herestraat 49, 3000 Leuven, Belgium; erik.vanherck@uzleuven.be; 8 International Agency for Research on Cancer, Dietary Exposure Assessment Group, 150 Cours Albert Thomas, 69372 Lyon CEDEX 08, France; HuybrechtsI@iarc.fr; 9 IDEWE, External Service for Prevention and Protection at Work, Interleuvenlaan 58, 3001 Heverlee, Belgium

**Keywords:** methyl-group donor, global DNA methylation, global DNA hydroxymethylation, pregnancy

## Abstract

It is still unclear to which extent methyl-group intake during pregnancy can affect maternal global DNA (hydroxyl)methylation. Pregnancy methylation profiling and its link with methyl-group intake in a healthy population could enhance our understanding of the development of pregnancy related disorders. One hundred forty-eight women were enrolled in the MANOE (MAternal Nutrition and Offspring’s Epigenome) study. Thiry-four women were enrolled before pregnancy and 116 during the first trimester of pregnancy. Global DNA (hydroxy)methylation in blood using LC-MS/MS and dietary methyl-group intake (methionine, folate, betaine, and choline) using a food-frequency questionnaire were estimated pre-pregnancy, during each trimester, and at delivery. Global DNA (hydroxy)methylation levels were highest pre-pregnancy and at weeks 18–22 of pregnancy. We observed a positive relation between folic acid and global DNA methylation (*p* = 0.04) and hydroxymethylation (*p* = 0.04). A high intake of methionine pre-pregnancy and in the first trimester showed lower (hydroxy)methylation percentage in weeks 11–13 and weeks 18–22, respectively. Choline and betaine intake in the first weeks was negatively associated with hydroxymethylation. Women with a high intake of these three methyl groups in the second and third trimester showed higher hyrdoxymethylation/methylation levels in the third trimester. To conclude, a time trend in DNA (hydroxy)methylation was found and women with higher methyl-group intake showed higher methylation in the third trimester, and not in earlier phases of pregnancy.

## 1. Introduction

**Figure 1:**
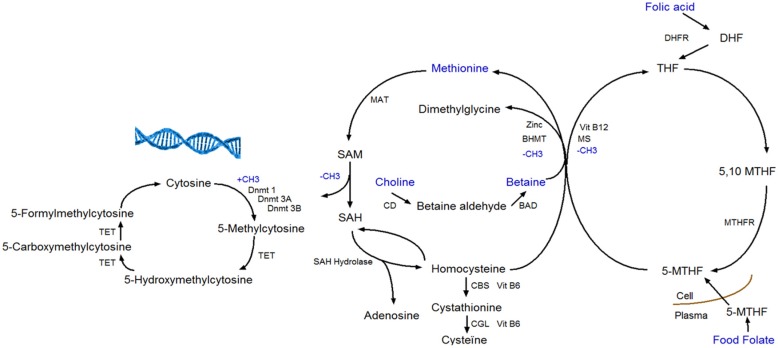
The one-carbon metabolism and DNA (hydroxy)methylation. BAD, Betaine Aldehyde Dehydrogenase; BHMT, Betaine Homocysteine Methyltransferase; CBS, Cystathionine Beta Synthase; CD, Choline Dehydrogenase; CGL, Cystathionine Gamma Lyase; DHF, dihydrofolate; DHFR, Dihydrofolate Reductase; Dnmt, DNA Methyltransferase; MAT, Methionine Adenosyltransferase; MS, Methionine Synthase; MTHF, Methyltetrahydrofolate; MTHFR, Methylenetetrahydrofolate Reductase; SAH, S-Adenosylhomocysteine; SAM, S-Adenosylmethionine; TET, Ten-eleven Translocation; and THF, Tetrahydrofolate.

A woman’s diet before and during pregnancy is important for the pregnancy’s outcome. It is a time period in which nutritional needs are higher because of the developing and growing fetus [1]. For instance, there is a higher need for methyl-group donors during pregnancy, like folate, betaine, choline, and methionine [2]. Folate is required for cell division because of its role in DNA synthesis. During pregnancy there is an increased folate demand due to fetal and uteroplacental organ growth. Dietary folate intake does not always meet the increased need; therefore, a daily folic acid supplement of 400 μg is recommended before and during the first three months of pregnancy [3]. Furthermore, there is also an increased demand of choline during pregnancy for the formation of new membranes [4]. Various pregnancy complications have been associated with folate and choline deficiency like neural tube defects [5,6]. The four methyl-group donors (methionine, folate, betaine, and choline) play a central role in the one-carbon metabolism (Figure 1). The one-carbon metabolism lies central in the methylation of all biological molecules, including DNA. Therefore, any dietary factor that influences this pathway may affect DNA methylation [7].

DNA methylation takes place at the carbon-5 position of cytosine in CpG (cytosine linked with a phosphodiester bond to guanine) dinucleotides when a methyl group (CH3) is added [8]. The transfer of a methyl group to the DNA depends on the availability of S-adenosylmethionine (SAM), which is derived from methionine in presence of methionine adenosyltransferase (MAT). SAM is further de-methylated by DNA methyltransferases to S-adenosylhomocysteine (SAH), which in its turn is hydrolyzed to homocysteine in a reversible reaction that strongly favors SAH synthesis. Homocysteine can leave the methionine cycle through the transsulfuration pathway or can be re-methylated to methionine through the methionine or folate cycle [2,9]. In the methionine cycle, the enzyme betaine homocysteine methyltransferase (BHMT) catalyzes the transfer of the methyl group from betaine in order to form both methionine and dimethylglycine. Betaine can be derived directly from the diet or converted from choline-containing foods [10]. In the folate cycle, folic acid and food folate are converted to 5-methyltransferase, which is the methyl-donor for the re-methylation of homocysteine to methionine [2,11].

Apart from DNA methylation to cytosine, 5-hydroxymethylcytosine (5-hmC) is a hydroxylated and methylated form of cytosine. The conversion of 5-methylcytosine (5-mC) to 5-hmC is considered being a step prior to demethylation. Ten-eleven translocation (TET) enzymes are responsible for the conversion of 5-mC to 5-hmC, and further to 5-formylcytosine (5-fC) and 5-carboxycytosine (5-caC), which are intermediates in the DNA demethylation pathway (Figure 1) [12]. Recent studies have examined the influence of dietary factors (e.g., vitamin C) on 5-hmC. Vitamin C not only induces increased levels of 5-hmC, but also of 5-fC and 5-caC in mouse embryonic stem cells [13].

During pregnancy, there are some critical windows of fetal development where dietary factors can influence the epigenome. For example, the epigenome is most vulnerable to environmental factors during embryogenesis because the DNA synthetic rate is high, and the DNA methylation patterns are established which are required for normal development and differentiation [14]. Nevertheless, it is still unclear to what extent the maternal intake of methyl-groups during pregnancy might affect maternal DNA methylation. Most of the studies that investigate the effect of methyl-group donor intake on DNA methylation do not assess diet at different time points during pregnancy and are limited to offspring DNA methylation. Dietary information should be obtained at several time points during pregnancy, because offspring DNA methylation and health risk is found to be different depending on the time of exposure during gestation [15]. Additionally, studies that assess maternal DNA methylation are mostly designed to identify pregnancy related disorders (preeclampsia and gestational diabetes mellitus) and risk of preterm birth. For instance, Anderson et al. [16] found a significant difference in first trimester DNA methylation sites of maternal white blood cells collected in pregnancies complicated by preeclampsia. Enquobahrie et al. [17] found that maternal DNA methylation in blood around 16 weeks of pregnancy was different in women who had two consecutive pregnancies in which only one pregnancy was complicated by gestational diabetes mellitus. Burris at al. [18] found that higher early pregnancy maternal LINE-1 methylation in white blood cells predicts lower risk of preterm birth. To date, to the very best of our knowledge, no studies have been carried out concerning the maternal DNA methylation pattern during pregnancy in a healthy population. Pregnancy DNA methylation profiling in a healthy population could enhance our understanding of the development of pregnancy-related disorders.

In this study, we aimed to determine whether there is a change in methyl-group donor intake and in the DNA (hydroxy)methylation pattern in maternal white blood cells during the course of pregnancy. Secondly, we determined the concurrent and previous (lagged) effect of maternal intake of methionine, folate, betaine, and choline on maternal global DNA methylation and hydroxymethylation pre-pregnancy, during each trimester of pregnancy, and at delivery in a Flemish (Belgium) population.

## 2. Materials and Methods

### 2.1. Study Subjects

**Figure 2:**
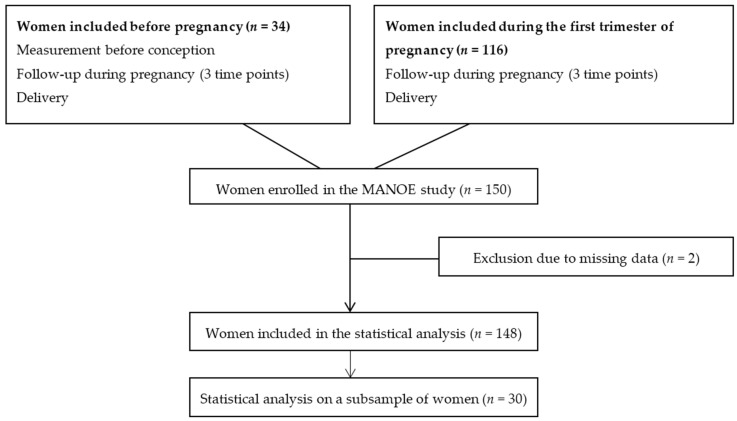
Flowchart of women enrolled in the MANOE study and included in the statistical analysis.

We studied participants enrolled in the MANOE (MAternal Nutrition and Offspring’s Epigenome) study, an ongoing prospective, observational cohort study initiated in April 2012. Healthy Caucasian women who desired to become pregnant or who were in the first trimester of pregnancy were recruited at the Department of Obstetrics and Gynecology of the University Hospitals Leuven (Belgium). We enrolled 150 women (34 women before pregnancy and 116 in the first trimester of pregnancy) between April 2012 and January 2015 (Figure 2). Exclusion criteria were: non-Caucasian women, multiple pregnancies (twins, triplets etc.), infertility treatment, and longitudinal missing data. Only two women had no methyl-group donor intake data and were not included in the statistical analysis, which gives us a total of 148 women (Figure 2). Fifteen of these 148 women developed pregnancy complications (gestational diabetes and preeclampsia) or delivered preterm. A screening for gestational diabetes was performed at 24–28 weeks using a 50 g glucose challenge test. When the test showed a glycaemia ≥140 mg/dL (≥7.8 mmol/L) a 75 g oral glucose tolerance test (OGTT) was also performed. Based on this test eight women were diagnosed with gestational diabetes mellitus (153–199 mg/dL or 8.5–11.0 mmol/L glucose) [19]. One woman was diagnosed with preeclampsia (systolic blood pressure > 140 mmHg and/or diastolic >90 mmHg with protein loss in the urine of >300 mg/24 h) and one had a high risk of neural tube defects and was, therefore, given an extremely high dose of folic acid (4 mg/day). Six infants were born preterm (<37 weeks of gestation).

This study was conducted according to the guidelines laid down in the Declaration of Helsinki and all procedures involving human subjects were approved by the UZ Leuven-Committee for Medical Ethics (ML7975). Written informed consent was obtained from all subjects.

### 2.2. Maternal Measurements

All 148 women were followed-up during pregnancy at their three scheduled ultrasounds (11–13 weeks, 18–22 weeks, and 30–34 weeks of gestation) and at delivery (Figure 2). From the women recruited before pregnancy (*n* = 34) we disposed of extra measurements before conception. At each time point we collected venous whole blood in 4.5 mL tubes containing ethylenediaminetetraacetic acid (EDTA) and in 5 mL serum separator tubes (BD Vacutainer Systems, 9320 Erembodegem, Belgium). From the women recruited before pregnancy, blood samples were taken the year before conception, and of all women blood samples were collected at the three ultrasounds and during delivery. We also dispose of a blood sample of the father and cord blood, however the paternal effect and effect on offspring methylation was not discussed in this paper. To assess maternal intake of dietary methyl-group donors (methionine, folate, betaine, and choline) before and during each trimester of pregnancy of women enrolled in the MANOE study a food-frequency questionnaire (FFQ) was developed and validated. We validated the FFQ in two validation studies (one using a food diary as reference method and one with additional biomarkers of the one-carbon metabolism) in women of reproductive age [20,21]. Ideally, food consumption data are linked to food composition data collected in the same region/country. Since, the Belgian food composition database NUBEL [22] does not contain information about the four methyl-group donors under study databases of neighboring countries were used in the validation of the FFQ. The Dutch NEVO food composition database [23] was used for folate and the German BLS Nutrient database [24] for methionine. For choline and betaine, no European database was available. However, the United States Department of Agriculture (USDA) database has a special section for the choline and betaine content of common foods. The total choline content was used and calculated as the sum of free choline, glycerophosphocholine, phosphocholine, phosphatidylcholine, and sphingomyelin [25]. The USDA database was also used for the nutrient content of folate and methionine if not found in NEVO and BSL databases, respectively. Some typically Belgian food products like “speculaas”, a type of shortcrust biscuit, are not included in the USDA database. For these products, the choline and betaine content of the American variety was entered in the database. For example, a ginger biscuit was used for the choline and betaine content of “speculaas”.

In each trimester, questions were asked about the use of nutritional supplements (frequency, brand/type, dosage). At the consultation in the first trimester (week 11–13) we asked women who were recruited during pregnancy about supplements intake before pregnancy (frequency, brand/type, dosage, and start before pregnancy). Only the intake of folic acid (synthetic form of folate) was registered, since there was no report on the supplemental intake of methionine, betaine, and choline. Furthermore, using a combination of questionnaires and interviews, we collected information about a range of socio-demographic factors, life style habits, and physical activity. A physical activity score was calculated before pregnancy and during each trimester of pregnancy by using a modified version of the Kaiser Physical Activity Survey. A total activity index was calculated as the sum of four activity indices (household/caregiving, occupational, active living, sports/exercise). Each activity index has a value that ranged from 1 to 5 representing increasing levels of participation [26]. Height and pre-pregnancy weight was used to calculate the Body Mass Index (BMI, kg/m^2^). Women included before pregnancy were weighed at the first consultation on a standard weighing scale (SECA Alpha model 888 or 877, Teleflex, Belgium) with indoor clothes (no shoes) to the nearest 0.1 kg. Pre-pregnancy weight of women included during pregnancy was self-reported. The height was measured without shoes with a microtoise to the nearest 0.5 cm (SECA model 206, Leicester Height Measure, Burmingham, UK). During each consultation women were weighed to calculate maternal gestational weight gain. Total gestational weight gain was calculated as the pre-pregnancy weight subtracted from the last clinically-recorded weight before delivery. Determination of gestational age was based on a crown rump length measured between 7 and 14 weeks of gestation in all patients [27].

### 2.3. DNA Methylation and Hydroxymethylation Measurements

Blood samples were put in the freezer (−20 °C) after collection and DNA was extracted from white blood cells with the salting out method [28]. The quantity and purity of DNA were determined by a NanoDrop. DNA of 150 women was analyzed by fast and sensitive liquid chromatography-tandem mass spectrometry (LC-MS/MS) method for the simultaneous quantification of 5-methylcytosine (5-mC) and 5-hydroxymethylcytosine (5-hmC) as described previously [29]. Briefly, isolated genomic DNA samples (10 μg) were hydrolyzed to individual deoxyribonucleosides by a simple one-step DNA hydrolysis procedure. For this, a digest mix was prepared by adding phosphodiesterase I, alkaline phosphatase and Benzonase Nuclease to Tris-HCl buffer. Extracted DNA was then hydrolyzed by adding 10 μL digest mix and incubating at 37 °C for at least 8 h. After hydrolysis, 490 μL of acetonitrile/water was added to each sample. Global DNA methylation and hydroxymethylation was obtained by quantifying 5-mdC, 5-hmdC, and dC using ultra-pressure liquid chromatography (UPLC), in combination with tandem mass spectrometry (MS-MS). Global DNA methylation was expressed as a percentage of 5-mdC versus the sum of 5-mdC, 5-hmdC and dC (%Global DNA methylation = 5-mdC/(5-mdC + 5-hmdC + dC)), while global DNA hydroxymethylation was expressed as a percentage of 5-hmdC versus the sum of 5-mdC, 5-hmdC and dC (%Global DNA Hydroxymethylation = 5-hmdC/(5-mdC + 5-hmdC + dC)).

### 2.4. Progesterone, Estradiol, and Total β-hCG Measurements

Serum was collected and stored at −80 °C. Serum samples of a subsample (30 women) were analyzed. Fifteen women with high (>percentile 75) and low (<percentile 25) intake of methyl-group donors were randomly selected. Samples were shipped to the laboratory (Algemeen Medisch Laboratorium, Antwerp, Belgium) where progesterone, estradiol, and total Beta-Human Chorionic Gonadotropin (β-hCG) in serum were measured using commercially available kits (Abbott Diagnostics, 1348 Ottignies/Louvain-La-Neuve, Belgium). ARCHITECT assays were used, which are Chemiluminescent Microparticle Immunoassays (CMIA) for the quantitative determination of hormones.

### 2.5. Statistical Analysis

First, we assessed the changes in intake of methyl-group donors and DNA (hydroxy)methylation before and during pregnancy using a multivariate regression model for longitudinal measurements with methyl-group donor intake and (hydroxy)methylation as a response variable and time point as a factor (Least Significant Difference (LSD) post hoc test). We have performed this analysis on all women (*n* = 148) and on women without pregnancy complications/pre-term delivery (*n* = 113). We also assessed the link between hormone level and DNA methylation status using a multivariate regression model for longitudinal measurements with DNA (hydroxy)methylation as a response variable, time point as a factor, and hormone level as a covariate. The analysis was done on the whole subsample (*n* = 30) and on the subsample without boxplot outliers (women with high/low values of (hydroxy)methylation and/or hormone levels) (*n* = 26). To determine the effect of maternal methyl-group donor intake on maternal global DNA (hydroxy)methylation during different time points, a multivariate regression model for longitudinal measurements with a time-varying covariate and lagged effects was used. Methylation and hydroxymethylation measurements were modeled as a function of methyl-group donor intake: at each time point, the model allows the influence of the concurrent nutrient intake value as well as the previous nutrient intake values (= lagged effects of nutrient intake). The model additionally allows the association between methyl-group donor intake and (hydroxy)methylation to be different between time points. All analyses were corrected for the presence of pregnancy complications, maternal age, pre-pregnancy BMI, smoking, and physical activity. These variables were found to be associated with maternal intake of methyl-group donors and DNA (hydroxy)methylation. An unstructured residual covariance matrix was modeled to account for clustering between longitudinal measurements. The methodology provided valid estimates under the plausible assumption that, given the observations, missing values did not depend on non-observed values. Dummy variables were used to deal with missing values by design, meaning that no previous measurement (lagged effect) was available for the first time point (pre-conception), or no concurrent measurement was available for the last measurement (delivery). Smoking and physical activity were registered at each time point. In case of missing observations on physical activity, the adjacent (earlier or later) level was considered for that time point. All tests were two-sided, a 5% significance level was assumed for all tests. Analyses were performed using SAS software (version 9.4 of the SAS System for Windows, 3080 Tervuren, Belgium).

## 3. Results

The characteristics of the women included in the study population are presented in Table 1. Mothers had a mean age of 30.63 years (SD 3.52), a mean BMI of 22.75 kg/m^2^ (SD 3.3), a mean gestation age of 39.41 weeks (SD 1.19), 48% were pregnant with their first child, and 52% had a university degree. 77.7% of the women took a folic acid supplement before conception and 98% during the first trimester of pregnancy. 4.73% of the women smoked before and in the first trimester of pregnancy, and some of these women (2.7%) continued smoking in the second and third trimester. The physical activity level was highest before pregnancy (10.76) and lowest in the third trimester (9.55).

The intake of methionine (*p* = 0.49), choline (*p* = 0.37), and betaine (*p* = 0.17) was stable and did not change over time during pregnancy (Table 2). However, there was a large variation in the intake of these 3 methyl-group donors between the women. For example, the minimum intake of methionine before pregnancy is 818.3 mg and a maximum intake of 2542.2 mg. Women with a high/low intake of these three methyl-group donors in the beginning of pregnancy also have a high/low intake later in gestation. Folate and folic acid intake changed significantly within the time frame of pregnancy. The intake of folate before pregnancy was significantly higher (298.4 μg/day) than the intake in the first trimester (10–13 weeks) (269.3 μg/day), the second trimester (18–22 weeks) (261.8 μg/day), and the third trimester (30–34 weeks) (268.2 μg/day) of pregnancy. The intake of folic acid on the other hand was the highest in the first trimester of the pregnancy (560.8 μg) and was significantly different from the intake at the other three time points (447.2 μg pre-pregnancy, 424.7 μg at 20 weeks, and 406.8 μg at 30 weeks).

**Figure 3:**
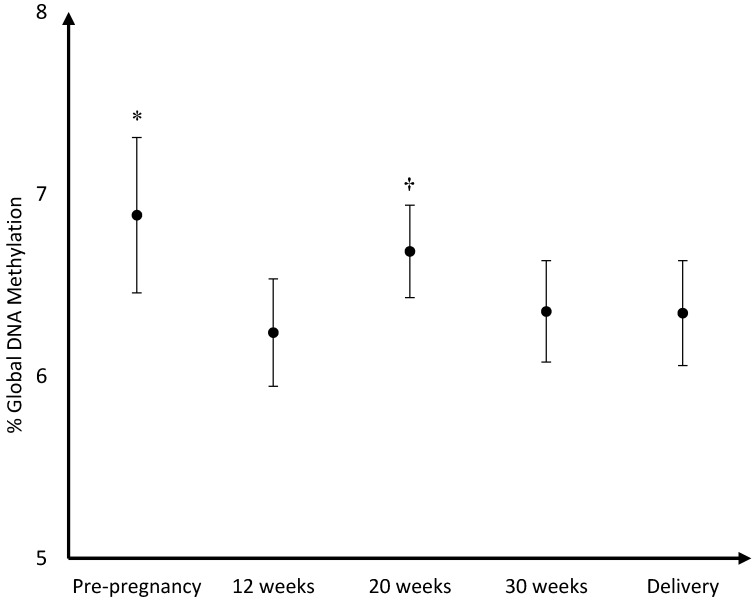
Mean DNA methylation percentage and 95%CI before pregnancy (*n* = 34), during pregnancy, and at delivery (*n* = 114); * %Global DNA methylation pre-pregnancy higher than %methylation at 12 weeks (*p* = 0.007), 30 weeks (*p* = 0.04), and at delivery (*p* = 0.04); † %Global DNA methylation at 20 weeks (6.69%) higher than %methylation at 12 weeks (*p* = 0.004), and 30 weeks (*p* = 0.04).

**Figure 4:**
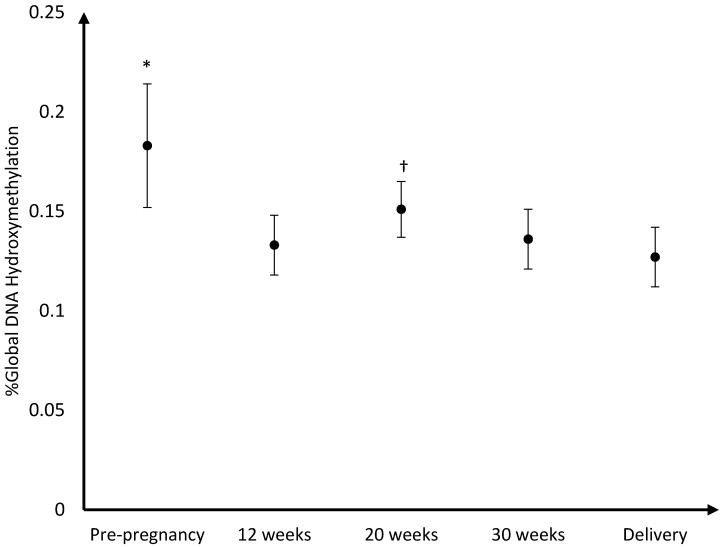
Mean DNA hydroxymethylation percentage and 95% CI before pregnancy (*n* = 34), during pregnancy, and at delivery (*n* = 114); * %Global DNA hydroxymethylation pre-pregnancy was higher than % hydroxymethylation at 12 weeks (*p* = 0.003), 30 weeks (*p* = 0.006), and at delivery (*p* = 0.002); † %Global DNA hydroxymethylation at 20 weeks was higher than %hydroxymethylation at 12 weeks (*p* = 0.046), and delivery (*p* = 0.02).

**Figure 5:**
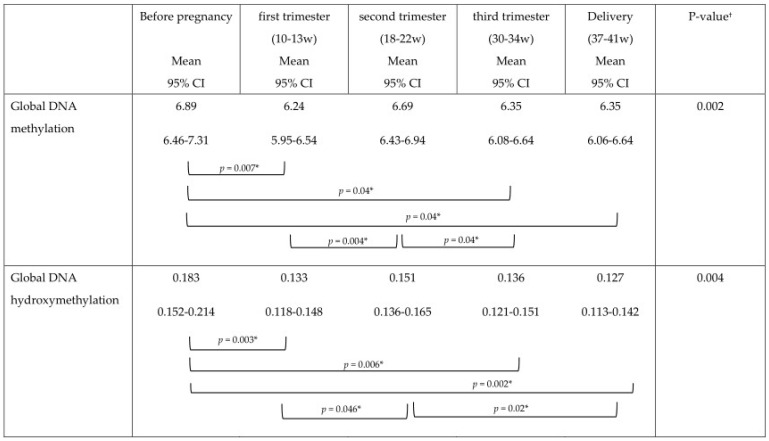
Mean DNA (hydroxy)methylation percentage and 95% CI before pregnancy (*n* = 34), during each trimester of pregnancy and at delivery (*n* = 114).

The mean DNA (hydroxy)methylation percentage before, and during each trimester of pregnancy, and at delivery is shown in Figure 3, Figure 4, Figure 5. The mean global DNA methylation percentage before pregnancy (6.89%) was significantly higher than the mean methylation percentage at 12 weeks (6.24%; *p* = 0.007), 30 weeks (6.36%; *p* = 0.04), and at delivery (6.35%; *p* = 0.04). During pregnancy, the mean global DNA methylation percentage was the highest at 20 weeks (6.69%) and was found higher than the mean methylation at 12 weeks (6.24%; *p* = 0.004), and 30 weeks (6.36%; *p* = 0.04). For hydroxymethylation the same results were observed. The mean DNA hydroxymethylation percentage before pregnancy (0.183%) was higher than the mean hydroxymethylation percentage at 12 weeks (0.133%; *p* = 0.003), 30 weeks (0.136%; *p* = 0.006), and at delivery (0.127%; *p* = 0.002). During pregnancy, the mean DNA hydroxymethylation percentage was the highest at 20 weeks (0.151%) and was significantly higher than the mean hydroxymethylation at 12 weeks (0.133%; *p* = 0.046), and delivery (0.127%; *p* = 0.02). Analysis of data from women without pregnancy complications/pre-term delivery (*n* = 113) gave similar results.

**Figure 6:**
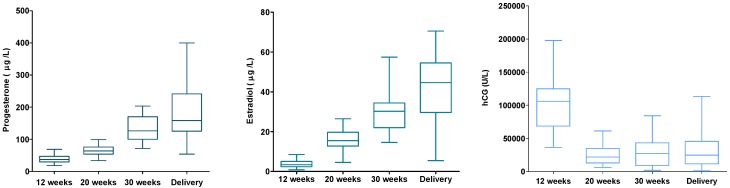
Maternal serum progesterone, estradiol, and β-hCG during pregnancy (*n* = 30).

From a subsample of 30 women, hormone levels of serum progesterone, estradiol, and β-hCG were analyzed (Figure 6). The hormone levels were linked to the women’s DNA (hydroxy)methylation status. No associations were found for these hormones and DNA (hydroxy)methylation. However, when excluding four women from the analysis, women with very high/low (hydroxy)methylation and/or hormone levels (outliers), higher β-hCG levels were associated with lower levels of global DNA methylation (*p* = 0.021).

The results of the concurrent and previous (lagged) effect of maternal methyl-group donor intake on maternal global DNA (hydroxy)methylation before and during pregnancy can be found in Table 3 and Table 4. There was no evidence of any association between concurrent dietary methyl-group donor intake (either in first, second, or third trimester) and DNA (hydroxy)methylation at the same time point (Table 3). For example the intake of methionine at 11–13 weeks was not associated with DNA methylation measurements at 11–13 weeks. In contrast, we observed a relation between supplemental intake of folic acid and DNA methylation (*p* = 0.043) and hydroxymethylation (*p* = 0.043). A higher supplemental intake of folic acid at a specific time point resulted in a higher DNA (hydroxy)methylation percentage at that same time point (Table 3). There was no evidence of a relation between previous nutrient methyl-group donor intake and DNA (hydroxy)methylation for folate and folic acid, and for DNA methylation also no relation with previous choline and betaine intake from food. There was a significant association between previous methionine intake and DNA (hydroxy)methylation, but it depended on the time point. At 18–22 weeks, there was a significant negative association (*p* = 0.05) between previous methionine intake (11–13 weeks) and DNA methylation, meaning that a higher intake of methionine at 11–13 weeks was associated with a lower methylation level at 18–22 weeks. At 30–34 weeks, there was a significant positive relation for DNA hydroxymethylation with previous (18–22 weeks) methionine (*p* = 0.009) and choline (*p* = 0.02) intake. Although significant interaction effects between DNA (hydroxy)methylation and methionine, choline, and betaine were found, only three significant p-values were observed at the different time points and no significant result at the different time points for betaine. The overall significant interaction effects were most likely caused by a change in trend in the middle of pregnancy. Negative slopes were observed in early pregnancy and positive slopes later in pregnancy. Negative slope values (in the beginning of pregnancy) indicated that a higher methionine, choline, and betaine intake before pregnancy and at 11–13 weeks of pregnancy was related with lower levels of DNA (hydroxy)methylation at 11–13 weeks and 18–22 weeks of pregnancy, respectively. Positive slope values (at the end of pregnancy) on the other hand indicated that a higher methionine, choline, and betaine intake at 18–22 and 30–34 weeks of pregnancy was related with higher levels of DNA (hydroxy)methylation at 30–34 weeks and delivery, respectively (Table 4).

## 4. Discussion

To our very best knowledge, this study was the first to show the maternal global DNA methylation and hydroxymethylation pattern during the course of pregnancy. The same trend was found for global DNA methylation and hydroxymethylation, which was to be expected since there is a close relationship between the two epigenetic processes. Indeed, Tellez-Plaza et al. reported that global DNA methylation and global DNA hydroxymethylation measured in blood were positively associated in a subsample of the Strong Heart Study (48 participants) [30]. For both global DNA methylation and hydroxymethylation the measurements were highest before pregnancy and in the second trimester (week 18–22) of the pregnancy. With some caution, we might say that the trend in the DNA (hydroxy)methylation pattern could be linked with hormonal changes during pregnancy. DNA (hydroxy)methylation was highest in the second trimester of pregnancy at which time the peak of β-hCG was passed and levels of estradiol and progesterone were still low.

We determined the concurrent and previous (lagged) effect of maternal intake of folate, betaine, choline, and methionine on maternal global DNA methylation and hydroxymethylation pre-pregnancy, during each trimester of pregnancy and at delivery. We found no evidence of an association between concurrent dietary methyl-group donor intake and global DNA (hydroxy)methylation. In the current cohort, the intake of dietary methionine, betaine, and choline was stable during the course of pregnancy, whereas intake of folic acid was the highest before (77.7%) and during the first trimester (98%) of pregnancy. Boeke at al. investigated the relation between gestational intake of methyl donor nutrients (betaine, choline, folate, and vitamin B12) and LINE-1 DNA methylation in first and second trimester maternal blood. They did not find any association between maternal methyl-group donor intake (periconceptionally and during the second trimester) and first and second trimester LINE-1 methylation [31]. A possible explanation for the lack of an association between dietary methyl-group donor intake and DNA (hydroxy)methylation is the use of a FFQ for dietary assessment, which likely introduced some measurement errors. We observed a positive association between the supplemental intake of folic acid and global DNA (hydroxy)methylation at each time point. Results from other studies are somewhat inconsistent. Folic acid supplementation of patients with colorectal adenoma for 10 weeks increased global DNA methylation [32], however a controlled folate depletion and repletion did not affect global DNA methylation in young Mexican American women [33]. The difference in response could be due to differences in age, duration, and magnitude of the supplementation. A possible explanation of the fact that only supplemental intake of folic acid had a direct effect on global DNA (hyrdoxy)methylation might be that folic acid has a higher bio-availability than food folate (almost 100% for folic acid vs. 50% for food folate) [34] or because we had more detailed information about the use of supplements before and during pregnancy which leads to a better estimation of the intake.

Despite the association of folic acid supplementation with global DNA (hydroxyl)methylation, there was no evidence of an association with previous nutrient folate and supplemental folic acid intake. Other studies found that there was a delay in DNA methylation response to folate intake. For example, Rampersaud et al. [35] reported that a significant proportion of elderly women during folate repletion showed a decrease in DNA methylation even though significant improvements were detected in blood folate indices. The delay in DNA methylation response to folate intake may be related, in part, to the very slow in vivo turnover of whole body folate pools [36]. In our cohort, most of the women took a folic acid supplement before and during pregnancy, so we can assume that if there was a delay in DNA methylation response, it would have happened before pregnancy.

Women with a high intake of methionine, choline, and betaine pre-pregnancy and in the first 12 weeks showed a lower (hydroxy)methylation percentage for methionine and a lower hydroxymethylation percentage for choline and betaine. A high intake of these three methyl-group donors in the second and third trimester of pregnancy was associated with higher (hyrdoxy)methylation levels for methionine and higher hydroxymethylation levels for choline and betaine in the third trimester and at delivery. A possible explanation is the shift in the one-carbon metabolism during pregnancy, which lies central in the methylation of the DNA. A higher rate of transsulfuration has been observed in early gestation and a higher rate of transmethylation in the third trimester [37]. In the transulfuration pathway, homocysteine leaves the methionine cycle by forming the amino acids cysteine and taurine. A higher rate of transulfuration in early gestation could explain why a high intake of methyl-group donors would result in lower maternal methylation. In the transmethylation pathway, homocysteine is re-methylated to methionine through the methionine or folate cycle. A higher intake of methyl-group donors in the third trimester could, therefore, result in higher methylation [9]. The change in trend in the midst of pregnancy was not found for folate and folic acid. A reason that the intake of folate was not associated with DNA (hydroxy)methylation could be that only 10%–20% of the methyl-group donors are derived from folate, 20% from methionine, and 60% from choline, highlighting the importance of the methionine cycle [38].

There are some strengths and limitations in the present study we need to address. The strengths of the present study include a unique study design that allowed us to collect longitudinal data starting before pregnancy, during each trimester of pregnancy, and at delivery. The use of a validated food-frequency questionnaire designed to assess the intake of the nutrients under study. Transposable element (LINE-1 and Alu) methylation is often used as a surrogate for global DNA methylation. The LC-MS/MS method used in this study can simultaneously quantify 5-methylcytosine (5-mC) and 5-hydroxymethylcytosine (5-hmC) (which is a novel and relatively unknown epigenetic mark). In addition, this method has a high specificity and sensitivity (low limit of detection). A first limitation is that we measured global DNA methylation and hydroxymethylation in white blood cells, which is composed of different cell types, each with a different DNA methylation profile. Additionally, we do not know how alterations of global DNA methylation in white blood cells reflect DNA methylation changes in other tissues. This is a problem in most human epidemiological studies. Another limitation is the fact that the Belgian food composition database NUBEL [22] does not contain information about the four methyl-group donors under study. Databases of neighboring countries or the USDA database for choline and betaine [25] content were used in the validation of the FFQ [20,21]. For folate, the Dutch NEVO food composition database was used [23] and the German BLS Nutrient database for methionine [24]. The USDA database was also used for the nutrient content of folate and methionine if not found in NEVO and BSL databases respectively. The limitations of using food composition databases from different countries include differences in description of foods, calculation of nutrient content, recipe calculations, etc.

## 5. Conclusions

We were able to detect a global DNA (hydroxy)methylation profile starting before pregnancy until delivery in a healthy population, with a higher DNA (hydroxy)methylation level before conception and at 20 weeks of pregnancy. An association was found with the intake of methyl-group donors through diet and supplements. We observed a positive relation between supplemental intake of folic acid and global DNA (hydroxy)methylation. Women with higher intake of nutritional methyl donors (methionine, betaine, and choline) before, and in the beginning of, pregnancy showed lower global DNA hydroxymethylation levels (and lower DNA methylation levels for methionine) in the first and second trimester. On the other hand, a higher intake in the second or third trimester resulted in higher DNA hydroxymethylation (and higher DNA methylation for methionine) in the third trimester and at delivery.

## 6. Future Research

Is there a “normal” maternal global DNA (hydroxy)methylation profile? The current manuscript describes the variation in DNA methylation before and across pregnancy. In future research, we would suggest to compare the global maternal DNA (hydroxy)methylation profile of pregnancies with and without pregnancy-related complications, e.g.,: women who delivered pre-term and at-term and women who developed gestational diabetes. This latter information could be used in a clinical setting to predict pregnant women’s risk to develop gestational diabetes. Furthermore, nutritional interventions in early pregnancy could prevent the occurrence of gestational diabetes later in gestation.

What about gene specific DNA methylation? In this study we firstly focused on the changes in global DNA (hydroxy)methylation before and during pregnancy. We observed global changes, but do not know which genes and biological processes were affected. Based on our current results, we would suggest analyzing genes related to the one-carbon metabolism or disease outcome (e.g., gestational diabetes). In a further study we will analyze some specific genes such as LEP using pyrosequencing.

Is there need to also assess offspring DNA methylation profiles? The newborn DNA methylation profile can be influenced by in utero experiences and methylation changes there, can be maintained into adulthood. Results from epidemiological mother-child cohorts will help to understand the impact of maternal environmental influences on maternal/offspring methylation and health.

## Figures and Tables

**Table 1:** Characteristics of the women included in the study (*n* = 148).

Characteristics	%	*n*
Parity		
0	48	71
1	39.2	58
2	11.5	17
3	1.4	2
Education		
High School	11.5	16
Higher Education	36.5	55
University	52	77
Folic acid use		
Before pregnancy	77.7	115
11–13 weeks	98	145
18–22 weeks	25	37
30–34 weeks	28.4	42
Smoking		
Before pregnancy	4.7	7
11–13 weeks	4.7	7
18–22 weeks	2.7	4
30–34 weeks	2.7	4
	Mean	Range
Physical activity (Activity Index Score)		
Before pregnancy	10.8 (1.5)	6.7–13.4
11–13 weeks	10.1 (1.5)	6.7–13.4
18–22 weeks	9.9 (1.5)	6.1–13.4
30–34 weeks	9.6 (1.1)	4.5–13.2
Weight gain (kg)		
11–13 weeks	1.6 (2.2)	−4.4–9.4
18–22 weeks	5.4 (3)	−2.8–15.1
30–34 weeks	10.7 (3.8)	1.4–26.5
Delivery	14.7 (4.4)	1.9–31

**Table 2:** Intake of methyl-groups through diet and supplements (folic acid) before (*n* = 34) and during pregnancy (*n* = 114).

	Time Point (Gestational Age)	Before Pregnancy Mean ± SE	First Trimester (10–13 Weeks) Mean ± SE	Second Trimester (18–22 Weeks) Mean ± SE	Third Trimester (30–34 Weeks) Mean ± SE	*p*
Methyl-Group Donor						
**Methionine (mg)**		1685.5 ± 64.3	1607.9 ± 41	1591.6 ± 41.2	1592.1 ± 45	0.49
**Folate (μg)**		298.4 ± 11.5	269.3 ± 7.5	261.8 ± 7.6	268.2 ± 8.9	0.01
**Folic acid (μg)**		447.2 ± 23.8	560.8 ± 40.5	424.7 ± 34.9	406.8 ± 36	<0.001
**Choline (mg)**		289.9 ± 10.4	269.2 ± 6.5	266.7 ± 6.6	269.7 ± 8	0.37
**Betaine (mg)**		175.8 ± 7.7	164 ± 4.9	169 ± 5.5	167.4 ± 5.7	0.17

*p*-values were obtained using a multivariate regression model for longitudinal measurements.

**Table 3:** Concurrent effect of methyl-group donor intake on DNA (hydroxy)methylation.

Concurrent Intake	DNA Methylation	DNA Hydroxymethylation
	p (Slope)	p (Slope)
	95% CI	95% CI
Methionine	0.7 (0.007)	0.83 (−0.0002)
	−0.03–0.04	−0.0023–0.0018
Folate	0.99 (0.001)	0.28 (−0.006)
	−0.2005–0.203	−0.02–0.005
Folic acid	0.04 (0.042)	0.04 (0.002)
	0.001–0.08	0.0001–0.004
Choline	0.62 (0.056)	0.89 (−0.001)
	−0.17–0.28	−0.0135–0.0117
Betaine	0.87 (0.023)	0.79 (−0.002)
	−0.26–0.31	−0.19–0.01

Slope> (<) 0: increase of nutrient level implies an increase (decrease) of the global DNA (hydroxyl)methylation level.

**Table 4:** Lagged effect of previous methyl-group donor intake on DNA (hydroxy)methylation.

Previous Intake	DNA Methylation	DNA Hydroxymethylation
	p (Slope)	p (Slope)
	95% CI	95% CI
**Methionine**	**0.03**	**0.003**
11–13 weeks	0.91 (0.007)	0.54 (−0.002)
	−0.11–0.13	−0.008–0.004
18–22 weeks	0.05 (−0.052)	0.06 (−0.003)
	−0.1–−0.001	−0.006–0.0002
30–34 weeks	0.15 (0.046)	0.009 (0.004)
	−0.02–0.11	0.001–0.007
Delivery	0.13 (0.053)	0.1 (0.003)
	−0.02–0.12	−0.0006–0.007
**Folate**	0.53 (−0.0006)	0.79 (0.000)
	−0.003–0.001	−0.0001–0.0001
**Folic acid**	0.84 (0.000)	0.88 (−0.000)
	−0.0003–0.0004	(−0.000–0.000)
**Choline**	0.79 (0.0003) −0.002–0.003	**0.02**
11–13 weeks		0.6 (−0.011)
		−0.05–0.03
18–22 weeks		0.16 (−0.014)
		−0.03–0.006
30–34 weeks		0.02 (0.024)
		0.003–0.04
Delivery		0.15 (0.015)
		−0.005–0.04
**Betaine**	0.86 (−0.0003) −0.0032–0.0027	**0.02**
11–13 weeks		0.08 (−0.048)
		−0.1–0.005
18–22 weeks		0.22 (−0.016)
		−0.04–0.01
30–34 weeks		0.11 (0.02)
		−0.005–0.05
Delivery		0.11 (0.02)
		−0.005–0.005

Slope> (<) 0: increase of nutrient level implies an increase (decrease) of the global DNA (hydroxy)methylation level.
